# The usefulness of growth hormone treatment for psychological status in young adult survivors of childhood leukaemia: an open-label study

**DOI:** 10.1186/1471-2431-8-25

**Published:** 2008-06-20

**Authors:** Jaap Huisman, Eline J Aukema, Jan Berend Deijen, Silvia CCM van Coeverden, Gertjan JL Kaspers, Heleen JH van der Pal, Henriette A Delemarre-van de Waal

**Affiliations:** 1 Department of Medical Psychology, VU University Medical Center, P.O. Box 7057, 1007 MB Amsterdam, The Netherlands; 2 Department of Clinical Neuropsychology, VU University, van der Boechorststraat 1, 1081 BT, Amsterdam, The Netherlands; 3 Department of Pediatrics, VU University Medical Center, P.O. Box 7057, 1007 MB Amsterdam, The Netherlands; 4 Department of Medical Oncology, Academic Medical Center, P.O. Box 22660, 1100 DD Amsterdam, The Netherlands; 5 Psychosocial Department, Emma Children's Hospital/Academic Medical Center, P.O. Box 22660, 1100 DD Amsterdam, The Netherlands; 6 Department of Public and Occupational Health, EMGO-institute, van der Boechorststraat 7, 1081 BT Amsterdam, The Netherlands

## Abstract

**Background:**

To reduce the risk of brain damage children with acute lymphoblastic leukaemia (ALL) are nowadays mainly treated with intrathecal chemotherapy (ITC) instead of central nervous system (CNS) radiation therapy (CRT) to prevent CNS relapse. However, chemotherapy may also lead to cognitive deficits. As growth hormone deficiency (GHD) or impaired growth hormone secretion are frequently found in ALL patients treated with cranial radiation therapy and/or chemotherapy, we hypothesized that GH therapy may reduce cognitive deficits in these patients.

**Methods:**

Twenty young adult survivors of childhood ALL with reduced bone mineral density (<-1 SD) and/or low IGF-I SD-scores (<-1 SD) were included in the study. A final group of 13 patients (9 males and 4 females), mean age 23.7 ± 2.9 years (range 20 – 29.7) completed a 2-year treatment with GH.

IQ and neuropsychological performance were assessed at pre-treatment (T1) and after one (T2) and two (T3) years. ANOVA was performed with assessment at T1, T2 and T3 as repeated measurements factor. Relations between test score changes and changes of IGF-I levels were determined by calculating the Pearson correlation coefficient.

**Results:**

Scores on the cognitive tests were in the normal range. Verbal short- and long-term memory performance decreased between T1 and T2, and increased between T2 and T3. Performance at T3 was not significantly different from that at T1. Performance for sustained attention improved from T1 to T2 and from T1 to T3. Visual-spatial memory was improved after one year of GH treatment. A significant positive correlation was found for Δ IGF-I (T2-T1) with difference scores of visual-spatial memory (T2-T1 and T3-T1), indicating that IGF-I increase after one year of GH treatment is associated with increase in cognitive-perceptual performance at month 12 and 24.

**Conclusion:**

Since the level of intellectual functioning of our patient cohort was in the normal range the present finding that GH treatment has negative effects on verbal memory and positive on attention and visual-spatial memory warrants similar studies in other groups of ALL survivors. Also, a lower dose of GH should be determined inducing as much IGF as needed to improve verbal as well as visual cognitive functions. The present findings indicate that more knowledge is needed before GH treatment may be recommended to enhance cognitive functions in ALL survivors.

## Background

In the last decades the prognosis of children with acute lymphoblastic leukaemia (ALL) has improved dramatically and long-term survival rates up to 80% have been reported [1]. Along with this development much research has been directed at identifying the psychological effects of treatment for ALL in childhood, in particular the neuropsychological sequelae. Prophylactic intrathecal (IT) chemotherapy (ITC) has replaced CNS radiation therapy (CRT), as research revealed cognitive deterioration and deficits associated with such CRT [2,3]. However, negative effects of high-dose IT chemotherapy regimens have been reported. For instance, children with ALL who had received chemotherapy for 3 years were more impaired on neurocognitive tasks involving right hemisphere simultaneous processing than their healthy siblings and than ALL children diagnosed recently or receiving chemotherapy for one year [4]. From a study by Copeland et al. [5] the cognitive side effects of IT chemotherapy appeared to be slightly more apparent 5 to 11 years after diagnosis than at 3-year follow-up. Since the differences were not clinically meaningful, the effects of chemotherapy in the absence of CRT were concluded to be slight. This is in accordance with the finding that the intellectual performance of survivors of paediatric ALL (age 6.8–33.7 years) treated with chemotherapy was normal [6]. In contrast, from thirty three reviewed studies on the long-term consequences of CNS chemotherapy in ALL survivors approximately two thirds document a decline in cognitive abilities [7]. For instance, patients treated with chemotherapy showed worse memory and fine-motor functions, but not school level, than their siblings [8]. Recently, behavior problems but also poor school performance was found in children with ALL attending primary school compared with same-age peers, although the rate of utilization of special education services was low. Thus, treatment for childhood ALL with chemotherapy may be associated with subtle but significant behavioral and educational problems [9].

Indeed, as with CRT, there is evidence that systemic CNS-directed and IT chemotherapy may lead to brain abnormalities, such as decreased cerebral perfusion, slower resting electroencephalogram frequencies, white matter changes and enlargement of the ventricles and cortical sulci [7].

From the studies cited above it may be concluded that cognitive impairment may be present in children with ALL who have been treated with CRT or IT chemotherapy. Therefore, a number of approaches to remediate these deficits, such as cognitive remediation, pharmacology and ecological alterations in the classroom, have been proposed. With respect to pharmacotherapy, in particular the use of phenylphenidate seems to be encouraging for ALL survivors with learning and attention problems [10].

Another pharmacological approach may be the use of growth hormone (GH) treatment. Growth hormone deficiency (GHD) or impaired GH secretion are frequently found late effects in patients treated with cranial radiation therapy and/or chemotherapy for childhood ALL [11-13].

Cognitive functioning and IQ appears subnormal in patients with GHD, as patients complain of lapses of attention, difficulty in concentrating, and forgetfulness. Moreover, their IQ score and educational level appear to be positively related to the concentration of insulin-like growth factor I (IGF-I), a serum marker for GH status, suggesting that subnormal cognitive performance is specifically related to GHD [14,15]. The association between cognitive functions and GH may be explained by the presence of many binding sites of GH and IGF-I in the hippocampus, a brain structure that is important for learning and memory functions [16]. Some studies suggest that GH therapy can have beneficial effects for the cognitive functioning of GH deficient adults, in particular memory function [17] and attention [18,19]. The psychological effects of GH therapy in a group of survivors of childhood cancer have been investigated only once before. However, aim of that study was to establish health related Quality of Life of survivors (most of them ALL), whether or not with GHD requiring GH treatment, while effects on neurocognitive functioning were not studied [20].

The present study is the first that evaluated the effects of GH on neurocognitive functioning of ALL survivors with a low bone mass and/or low IGF-I levels. We hypothesized that GH therapy would show positive effects on neurocognitive functioning, in particular attention and memory, in adult survivors of paediatric ALL.

## Methods

### Patients

Young adult survivors of childhood leukaemia with reduced bone mineral density (<-1 SD) and/or low IGF-I SD-scores (<-1 SD) were selected from a group of patients, treated for ALL between 1972 and 1990 at the paediatric departments of the VU University Medical Center and the Academic Medical Center in Amsterdam. The inclusion criteria were chosen like this because originally the study was designed to see whether bone mineral density would improve after GH intervention. Twenty out of 56 (36%) childhood ALL survivors over 20 years of age were included according to these criteria. These patients had never been tested for GHD and as a consequence were never treated with GH before.

Treatment of ALL had been given according to different treatment protocols. In general, treatment consisted of an intensive induction period, followed by a 2 to 3 year maintenance period. All patients received systemic cytostatic treatment, including corticosteroids, vincristine, methotrexate and 6-mercaptopurine and sometimes other cytostatic drugs were added. In addition, 17 patients out of the 20 included received prophylactic cranial irradiation in doses varying from 2000 to 2500 cGy. Three patients received high-dose methotrexate and intrathecal chemotherapy (methrotrexate, cytosine-arabinoside, prednison) instead of cranial irradiation to prevent central nervous system relapse.

**Figure 1:**
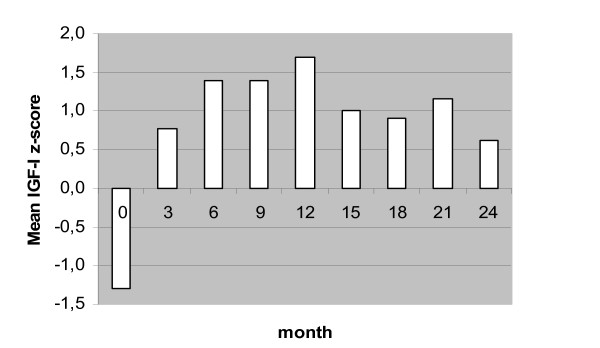
Mean IGF-1 Z-score at baseline (T1) and after 3, 6, 9, 12 (T2), 15, 18, 21 and 24 (T3) months of GH therapy.

ALL survivors were reviewed for age at diagnosis, treatment protocol, dose of irradiation, and time elapsed since irradiation. After an overnight fast a physical examination was performed and a blood sample was taken to assess IGF-I serum values. Blood samples were taken and IGF-I determined at baseline and at month 3, 6, 9, 12, 15, 18, 21 and 24. Mean IGF-I z-scores are shown in Fig. 1. Serum IGF-I was measured (in duplicate) after extraction by immunoradiometric assay (DSL, Webster, Texas USA). In addition, a provocative GH test was performed after overnight fast in all participants, using growth hormone releasing hormone in a dose of 100 μg intravenously (GHRH-test). To assess GH levels, blood samples were drawn at -15, 0, 10, 20, 30, 45, 60 and 90 minutes. The subjects rested during the test.

At another morning the insulin tolerance test (ITT) was carried out in the ALL survivors. Intravenous soluble insulin (Velosulin) was administered in a dose of 0.1 IE insulin/kg body weight. To assess GH levels, blood samples were taken at -15, 0, 10, 20, 30, 45, 60 and 90 minutes. During the ITT all subjects achieved blood glucose levels of < 2.0 mmol/l and there were clinical symptoms of hypoglycaemia as well. Blood glucose levels were measured every five minutes during the test.

The study protocol has been approved by the Medical Committee of Ethics on Human Research of the VU University Medical Centre and written informed consent was obtained from all patients.

### Procedure

#### Diagnosis of GH deficiency

The diagnosis of GHD was based on a GH peak response less than 9.0 mU/L (3 microgram/l) in both provocative tests. Four patients showed a GH peak response below 9.0 mU/L (3 microgram/l) for the ITT, but not for the GHRH-test; those patients were also considered growth hormone deficient. In this way, 13 out of 20 patients were GHD. Serum GH was measured in duplicate by immunometric assay (Sorin Biomedica, Saluggia Italy). The standards used were calibrated against the 1st IS 80/505 reference preparation.

#### Growth hormone therapy

Of the 20 ALL survivors with decreased BMD and/or IGF-I, three patients did not start with GH, because one of them was diagnosed with anorexia nervosa and the other two refused treatment. The remaining 17 patients started with GH-therapy for a two-year period (10 males, 7 females; mean age ± SD, 24.4 ± 3.3; range 20 – 31 yrs). Four patients stopped GH-therapy: two of them got pregnant within 3 months, one patient turned out to be growth hormone resistant and the other stopped for unknown reasons. A final group of thirteen participants (9 males and 4 females) completed the study. These participants were treated with growth hormone during 2 years. Four of them were not GHD (GH peak response in ITT between 11.0 and 70.0 mU/l). Mean age at diagnosis was 7.0 ± 4.3 years (range 2.5 – 15.2 years). Mean age at the start of the study was 23.7 ± 2.9 years (range 20 – 29.7 years). Mean IGF-1 (± SD) at the start of the study was 17.5 ± 5.3, and for IGF-Z-scores it was -1.3 ± 0.6 SD.

All patients used Humatrope subcutaneously with the Eli Lilly penfill system. The starting dose of GH was calculated as 0.1 mg per square meters of body surface. Every 2 weeks, the dose was increased with 0.1 mg/m2, until IGF-I rised above 0 SD. The GH dose that was needed to reach a level of IGF-1 of +1 SD according to sex and age was very different per individual. In the first 3 months, patients were seen after 2, 4, 8 and 12 weeks, after that every 3 months.

#### Assessment of (neuro)psychological functions

Participants were tested 3 times: before the start of GH treatment (T1) and after 1 and 2 years of treatment (T2, T3). Assessments were carried out by a well-trained and qualified test-assistant. All tests employed were standardized and do have documented data about reliability and validity.

#### Intelligence (assessment at T1)

Patients' IQ score (Full scale (FS)IQ, Performal (P)IQ and Verbal (V)IQ) was assessed only at T1 with the Dutch version of the Wechsler Adult Intelligence Scale (W.A.I.S.) [21].

#### Memory (assessments at T1, T2, T3)

- *15 Word Test*. Auditory verbal learning was tested in two conditions: immediate recall and recall after a 20-minute delay [22]. Two comparable versions were used (A at T1 and T3; B at T2) in order to minimize test-retest effects.

- *Benton Visual Retention Test*. In this test geometrical figures are presented and have to be recalled immediately by drawing. The test assesses visual memory, visual perception and visual-constructional capacities [23].

- *Digit Span *(subtest of WAIS). Digits are presented verbally and have to be recalled forward or reversed. Span of immediate verbal recall is tested.

- *Rey-Osterrieth Complex Figure Test*. After copy drawing of a complex geometrical figure subjects are asked to recall this drawing after 20 minutes. Visual-spatial memory is assessed [24]. To avoid test-retest effects we used two different figures: the Rey-Osterrieth figure at T1 and T3, and the Taylor figure at T2.

#### Attention (assessments at T1, T2, T3)

- *Fepsy (Auditory Reaction Time) *[25]. Auditory reaction time was assessed with the Auditory Reaction Time subtest, part of the Fepsy, a Dutch automated computerized test for cognitive functions. In this task the computer generates a 800 HZ auditory stimulus and the subject has to produce as quick as possible a push-button response. Reaction time for the dominant and the non-dominant hands are measured.

-*The Brickenkamp d2 test *measuring sustained attention. In this test a person is asked to detect and encircle correct letter-quotation mark-combinations. Total score is the number of correctly encircled letters minus the number of incorrect responses [26].

#### Executive functions (assessments at T1, T2, T3)

- *Trailmaking Test (A and B)*. Encircled numbers have to be connected consecutively by drawing lines (part A), or encircled letters and numbers have to be connected consecutively by alternating letters and numbers (part B). This test examines visual conceptual and visuomotor tracking [27].

### Data analysis

Scores at T1 were compared to test norms (independent samples test). Statistical analysis comprised analyses of variance (ANOVA) and correlation analyses. To determine the effects of GH therapy ANOVA was used with assessment at T1, T2 and T3 as repeated measurements factor. If the factor assessment yielded significant main effects within-subjects contrasts were evaluated. To determine relations between test score changes and changes of IGF-I levels the Pearson correlation coefficient was calculated. Significance level was defined as *p *≤ .05 (one-tailed). Data were analysed using the SPSS version 11 software package (SPSS inc., Chicago, USA).

## Results

Table 1 presents test scores prior to the start of GH treatment. The level of intellectual functioning (FSIQ, VIQ, PIQ) of the group survivors was high average. Memory tasks all resulted in test scores in the normal range of the population norms. Visual construction and visual motor tracking scores also were average in comparison to test norms. Sustained attention scores and auditory reaction time (dominant and non-dominant hand) were in the normal range as well.

**Table 1:** Mean pre-treatment (T1) scores ± SD of ALL survivors (n = 13)

Full scale IQ	112 ± 12	
Verbal IQ	110 ± 13	
Performal IQ	113 ± 13	High average
*Verbal short term memory*		
15 Word Test (number correct)	45.8 ± 3.8	Average
*Verbal long term memory*		
15 Word Test Recall (number correct)	9.2 ± 1.8	Average
*Visual-spatial short term memory*		
Benton (number correct)	8.2 ± 1.8	Average
*Short tem memory*		
Digit Span (WAIS) (number correct)	7.8 ± 3.6	High average
*Visual-spatial construction ability*		
Rey (copy) (number correct)	34.92 ± 1.5	
Rey (LTM)(number correct)	22.8 ± 6.3	Average
*Auditory RT*		
Fepsy dominant hand (ms)	242 ± 54.6	
Fepsy non-dominant hand (ms)	239 ± 56.9	Average
*sustained attention*		
D2 (number correct)	378 ± 88.7	Average
*Visual scanning and tracking*		
Trailmaking A (sec)	30.8 ± 10.9	
Trailmaking B (sec)	61 ± 16.9	Average

Mean scores at T1 ± SD; mean IQ = 100, SD = 15; higher test scores indicate better performance except scores in time (Auditory RT and Visual Scanning)

Changes in IGF-I Z-score levels across the treatment period are presented in Fig. 1. Levels at T2 and T3 were significantly higher than at baseline (*F*(1,12) = 39.1, *p *≤ .0005, *η*^2 ^= 0.76 and *F*(1,24) = 29.5, *p *≤ .0005, *η*^2 ^= 0.71, respectively). A significant decrease in IGF-I was seen between T2 and T3 (*F*(12,24) = 4.6, *p *= 0.05, *η*^2 ^= 0.28).

ANOVA showed significant differences between test sessions for Verbal short term memory (STM), Verbal long term memory (LTM), Visual-spatial long term memory and Sustained attention (see Table 2).

**Table 2:** Mean cognitive scores ± SD and significance levels of factor Session

	**Session**			**p-value (session)**		
	1	2	3	1–2	2–3	1–3

Verbal short-term memory	46 ± 3.8	40 ± 9.5	49 ± 6.9	**0.03**	**0.000**	0.14
Verbal long-term memory	9.2 ± 1.8	6.4 ± 3.0	9.6 ± 2.8	**0.008**	**0.000**	0.41
Visual-spatial long-term memory	22.8 ± 6.3	28.2 ± 4.5	26.0 ± 3.9	**0.004**	**0.05**	**0.07**
Sustained attention	378 ± 89	421 ± 100	435 ± 102	**0.02**	0.45	**<0.002**

Higher test scores indicate better performance

**Figure 2:**
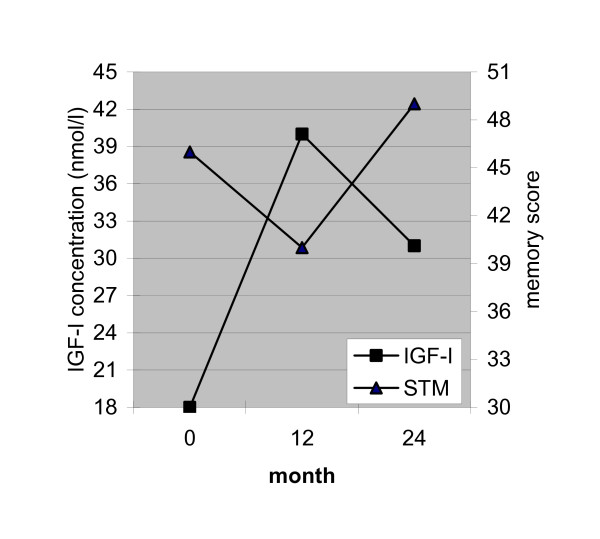
Mean IGF-I levels (nmol/l) and Verbal short term memory scores (15 Word Test – immediate recall) prior to start of GH treatment and after 12 and 24 months.

With respect to verbal short- and long-term memory a significant decrease in performance is seen between T1 and T2, followed by an increase between T2 and T3. The performance on verbal STM and verbal LTM is not significantly different between T1 and T3. As is clearly depicted in Fig. 2, the increase in IGF-I between T1 and T2 is accompanied by a decrease in short-term memory performance, while the decrease in IGF-I between T2 and T3 is accompanied by an increase in short-term memory scores. Exactly the same association with IGF-I was found for long-term memory.

Results for visual-spatial long term memory show an increase between T1 and T2 followed by a decrease between T2 and T3 (see table 2). The performance for visual-spatial long term memory at T3 only tends to be higher than at T1 (*p *= 0.07). An increase in performance for sustained attention is seen between T1 and T2. In addition, the performance at T3 is significantly better than at T1 (*p *< 0.002, see table 2).

As IGF-I levels were higher at T2 and T3 than at baseline we calculated the correlation between T2-T1 and T3-T1 difference scores of IGF-I and cognitive test scores. Significant positive correlations were only found of Δ IGF-I (T2-T1) with Δ visual-spatial long term memory (T2-T1) and with Δ visual-spatial long term memory (T3-T1) (*r *= 0.58, *p *= 0.02 and *r *= 0.53, *p *= 0.04, respectively). This means that IGF-I increase after one year of GH treatment is associated with increase in cognitive performance at month 12 and 24 relative to baseline. There was no correlation between Δ IGF-I (T3-T1) with Δ visual-spatial long term memory (T3-T1).

## Discussion

In literature the neurocognitive late effects of the treatment of childhood leukaemia have been studied extensively. Deleterious effects of treatment regimens including cranial radiation in particular have been documented. Results of studies on the effects of prophylactic CNS treatment with high-dose and intrathecal chemotherapy however show that such therapy also may have deleterious effects. Key question in the present study was whether GH treatment improves neurocognitive functioning of adult survivors of childhood leukemia. We hypothesized positive effects of GH treatment, in particular improvement of attention and memory, based on effect studies in growth hormone deficient adults [17-19].

In the present study a group of 13 young adult survivors of childhood leukaemia was assessed neuropsychologically before and during GH therapy installed because of decreased BMD and/or too low IGF-I. Most persons (10 of 13) had been treated with regimens including prophylactic cranial irradiation. Many years after treatment (mean time since diagnosis approximately 15 years) these survivors showed test scores on a variety of neuropsychological tasks in the normal range. Mean level of intellectual functioning as determined by IQ tests was even high average. This level is higher than expected, since most participants received prophylactic cranial irradiation as part of their ALL-treatments. The same holds for the test scores relating to specific neurocognitive functions we measured. Among others deficits in memory, visuospatial/motor skills and attention have been reported consistently in literature [7,28,29].

Although all available survivors meeting the growth and age criteria were invited to participate in this study and a high percentage (85%) agreed to do so, our small study group may be a quite selected sample not reflecting the cognitive functioning of the average population of ALL survivors. Because we have no information of pre-morbid functioning of these patients, and since our study lacks a control group, we can not exclude general or individual detrimental effects of ALL-treatment. Further, the observed high average IQ scores may be skewed due to the Flynn effect, that is Dutch test norms of the WAIS were published in 1970 and gains of IQ-points over time can add up from 2 to 3 points per decade [30]. However, if we adjust the mean high average IQ scores in the present patient group for the Flynn effect, these scores would be still in the normal range. This is in accordance with Von der Weid et al. [6] who described normal cognitive outcome in adolescent and young adult survivors of ALL. These survivors, however, were treated with chemotherapy alone. Since literature suggests different neurocognitive outcome in children with ALL treated with or without CNS irradiation and in view of our controversial findings concerning scores of intellectual and specific neuropsychological tests in adult survivors, the need for further investigation of the very long term neurocognitive effects of treatment of childhood ALL, with or without cranial irradiation, in adult survivors is underlined.

In the present study IGF-I levels were used as expression of neuroendocrine effects of growth hormone therapy and were related to testscores. Mean IGF-I level increased in the first year of treatment, decreased in the second year, but remained higher than at pre-treatment. After one year of GH treatment scores on tests measuring verbal short- and long-term memory decreased as IGF-I levels increased. After the second year of treatment the short- and long-term memory scores increased to the levels at T1 as IGF-I decreased. Thus, with respect to memory, an increase in IGF-I in the first year of treatment seems to be associated with impaired memory performance, which impairment seems to be counteracted by a reduced IGF-I level. In addition, sustained attention was significantly improved after the first and second treatment year, as IGF-I was highly increased. The absence of a difference in response on sustained attention between session 2 and 3 may be associated with the reduced IGF-I levels after one year of GH treatment. All in all, the GH-induced IGF-I increase in the first treatment year seems to impair specific (verbal) memory functions, which conclusion may be inferred from the observation that memory impairment is halted when IGF-I levels are lowered. It may well be true that the IGF-I increase in the fist year is too high to improve verbal memory functions which even results in an opposite effect, that is impairment of verbal memory. An other possible explanation could be that IGF-1 sensitivity of the brain is altered by prophylactic CRT.

Positive effects found in the present study concerns visual-spatial long term memory and sustained attention, which functions are improved after one year of treatment and respectively tend to be or are still improved after the second treatment year. Correlation analysis indicated that the IGF-I increase in the first treatment year is associated with an improvement of visual-spatial long term memory at month 12 and 24 relative to baseline. No relation was found between the increase in IGF-I during two years of treatment and changes in visual-spatial long term memory. As perceptual skills play a role in both spatial long-term memory and sustained attention, this result suggests that especially cognitive perceptual performance after one and two years of GH treatment are related to the IGF-I increase in the first year. Indeed, a relationship between IGF-I levels and perceptual functions has been reported by Aleman et al. [31]. In healthy male subjects (mean age of 69 years) higher IGF-1 levels were associated to better scores on the Digit Symbol Substitution test (W.A.I.S.), which test measures perceptual-motor functions and visual-spatial memory. As visual-spatial memory plays an important role in the performance on the Rey complex figure test it may well be true that a substantial increase in IGF-I may enhance visual-spatial (memory) functions.

We conclude that the present results show some beneficial effects of GH treatment on visual-spatial (long term) memory functions and attention in ALL survivors, while verbal memory functions are negatively affected.

## Conclusion

This study is the first presenting data about the neurocognitive functioning of adult survivors of childhood leukaemia in relation to GH treatment. Since most participants were treated with regimens including prophylactic cranial irradiation, the finding that these survivors showed at least an average level of intellectual functioning and neuropsychological test scores in the normal range is unexpected and asks for further research. The present finding that GH treatment has negative effects on verbal memory and positive effects on visual-spatial memory and attention warrants similar studies in other groups of ALL survivors. However, because of the unexpected high level of cognitive functioning and low levels of IGF-1 of our sample care is needed to generalize our results to the ALL population as a whole. In addition, the average baseline levels of cognitive functioning in our study group and positive effects of GH treatment on two particular neuropsychological tests while effects on other tests were absent or negative, questions clinical relevance. Further, because of these different effects of GH treatment on the neuropsychological tests observed positive effects may result from chance. In addition to the possibility that GH treatment may help ALL survivors with worse intellectual functioning, a lower dose of GH may be more effective as an increase too high of IGF-I may adversely affect verbal memory performance. However, it will be difficult to precisely determine the effective dose to improve verbal as well as visual cognitive functions, because a dose too low may in turn not affect *visual *memory and attention.

All in all, conclusions from studies in younger groups of survivors on neurocognitive toxicity of leukemia treatment, may not hold in all cases of young adult survivors. In addition, as has been described before in growth hormone deficient adults [17-19], the results of the present study suggest that relationships between GH therapy and aspects of neuropsychological functioning are strongly dependent on IGF-I levels. For theoretical and clinical reasons replication of this study and further research into these complex relationships is strongly recommended. Based on the present results, we conclude that more knowledge is needed before GH treatment to enhance cognitive functions in ALL survivors may be recommended.

## Competing interests

The authors declare that they have no competing interests.

## Authors' contributions

JH and EJA made substantial contributions to conception and design, acquisition, analysis, and interpretation of data and writing the manuscript. JBD was involved in analysis, interpretation of data and writing the manuscript. SCCMvC, GJLK, HJHvdP and HAD–vdW made substantial contributions to conception and design of the study. All authors read and approved the final manuscript.

## Pre-publication history

The pre-publication history for this paper can be accessed here:


